# Role of the Endocannabinoid System in Fibromyalgia

**DOI:** 10.3390/cimb47040230

**Published:** 2025-03-27

**Authors:** Mario García-Domínguez

**Affiliations:** 1 Program of Immunology and Immunotherapy, CIMA-Universidad de Navarra, 31008 Pamplona, Spain; mgdom@unav.es; 2 Department of Immunology and Immunotherapy, Clínica Universidad de Navarra, 31008 Pamplona, Spain; 3 Centro de Investigación Biomédica en Red de Cáncer (CIBERONC), 28029 Madrid, Spain

**Keywords:** endocannabinoid system, fibromyalgia, retrograde synaptic messenger, cannabinoid receptors, tender points, fibrofog

## Abstract

Fibromyalgia is a chronic disease marked by extensive musculoskeletal pain, persistent fatigue, and cognitive impairments. Despite its high prevalence, the underlying pathological mechanisms of fibromyalgia are still not fully elucidated. Emerging research has identified the endocannabinoid system as an essential factor in modulating pain and other symptoms related to fibromyalgia. The endocannabinoid system plays a key role in many physiological processes such as pain perception, mood regulation, and inflammation. This review provides a powerful analysis of the principal aspects of fibromyalgia and examines the evidence regarding the involvement of the endocannabinoid system in this condition, focusing on its influence on pain modulation. Moreover, the dysregulation of the endocannabinoid system in fibromyalgia patients will be examined, with an assessment of how variations in endocannabinoid levels and receptor activity may contribute to the clinical manifestations of the condition. A better knowledge of this physiological system could lead to the development of novel strategies for managing fibromyalgia.

## 1. Introduction

Fibromyalgia is a chronic pain syndrome characterized by musculoskeletal pain accompanied by several non-specific signs and symptoms, such as persistent fatigue, sleep disturbances, and cognitive impairments (like memory loss and difficulties with concentration) [1]. Pain associated with fibromyalgia can vary in both intensity and location and is frequently linked to sensitivity in particular areas referred to as tender points, as defined by the American College of Rheumatology (ACR) [2]. This condition affects 6.4% of the population in the United States of America, while in Europe and South America, its prevalence varies from 2.4% to 3.3% [3,4,5], with a higher prevalence observed in women [6]. Although the exact cause of fibromyalgia has not yet been clearly determined, it is typically believed to result from a combination of genetic and environmental factors [7].

Recent studies have identified the endocannabinoid system (ECS) as a critical component in the development of fibromyalgia [8]. The ECS, discovered in the 1990s, represents a sophisticated cell-signaling network crucial for maintaining homeostasis across numerous bodily systems [9]. This system is composed of three components: endocannabinoids, cannabinoid receptors, and the enzymes responsible for their synthesis and degradation. Endocannabinoids, primarily represented by anandamide (AEA) and 2-arachidonoylglycerol (2-AG), interact with cannabinoid receptors and are vital for managing numerous physiological processes, like pain perception, mood, appetite, memory, and inflammation [10]. Cannabinoid receptors are categorized into two main types: CB_1_R (predominantly located in the central nervous system—CNS—and play a key role in modulating pain perception, mood, and cognitive functions) [11,12] and CB_2_R (mainly situated in peripheral tissues and are involved in immune responses) [11,13]. Finally, the ECS includes synthesizing enzymes (diacylglycerol lipase—DAGL—or N-acyl phosphatidylethanolamine phospholipase D—NAPE-PLD) and degrading enzymes (fatty acid amide hydrolase—FAAH—and monoacylglycerol lipase—MAGL) [14].

Research indicates that alterations in endocannabinoid levels and receptor activity may contribute to the increased pain sensitivity and other symptoms observed in patients with fibromyalgia [8]. Given the role of the ECS, investigating its dysfunction in fibromyalgia could uncover new therapeutic approaches [8]. Targeting the ECS with pharmacological agents that modulate receptor activity or endocannabinoid levels provides a promising approach for improving the quality of life for patients with fibromyalgia [15]. Moreover, this insight underscores the vital role of the ECS in the complex pathology of fibromyalgia and underscores the necessity for further research into effective ECS-targeted therapies [15].

This article offers a comprehensive analysis of the ECS and its involvement in fibromyalgia. It explores the fundamental principles of the ECS, the potential role of its dysregulation in the symptoms of fibromyalgia, and the wider implications for managing this disease. By clarifying the interactions between the ECS and fibromyalgia, this article aims to enhance our comprehension of the disorder, support the creation of novel treatments, and advance patient outcomes.

## 2. Overview of the Endocannabinoid System

Research into the ECS commenced in the 1960s after THC (tetrahydrocannabinol) was discovered as the main psychoactive element of *Cannabis sativa* [16]. By contrast, cannabinoid receptors were revealed as members of the G protein-coupled receptor (GPCR) family. CB_1_R was the first receptor to be identified in the rat brain [17], followed by CB_2_R, which was initially recognized in a human leukemia cell line [18] but is also expressed in lymphocytes and the spleen [19,20]. The identification of cannabinoid receptors contributed to the discovery of endogenous molecules termed endocannabinoids, which interact with these GPCRs [21]. Research focused on identifying endogenous ligands has resulted in the discovery of two endocannabinoids: AEA and 2-AG. In summary, the ECS is a physiological system that encompasses cannabinoid receptors (CB_1_R and CB_2_R), their ligands (AEA and 2-AG), and the enzymes that control their synthesis and degradation [9].

### 2.1. Endocannabinoid Synthesis

**Figure 1:**
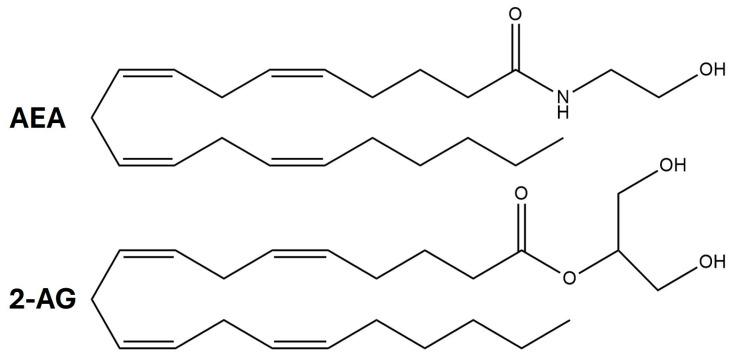
Chemical structures of AEA (anandamide) and 2-AG (2-arachidonoylglycerol).

**Figure 2:**
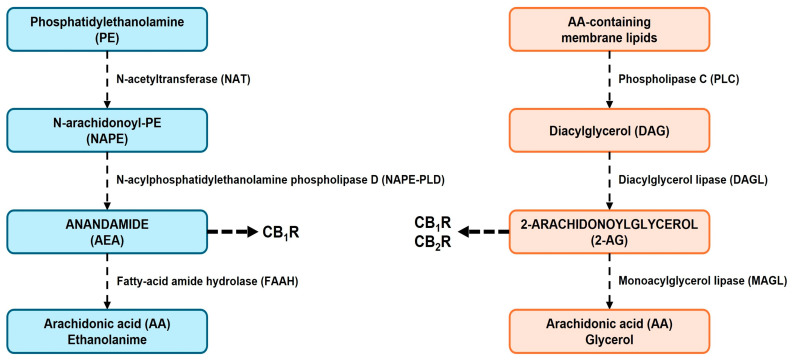
Overview of the biosynthesis of principal endocannabinoids (AEA and 2-AG) in several CNS regions, such as nucleus accumbens. Abbreviations: PE (phosphatidylethanolamine); NAT (N-acetyltransferase); NAPE (N-acylphosphatidylethanolamine); NAPE-PLD (N-acyl phosphatidylethanolamine-specific phospholipase D); AEA (anandamide); AA (arachidonic acid); FAAH (fatty-acid amide hydrolase); MAGL (monoacylglycerol lipase); 2-AG (2-arachidonoylglycerol); DAGL (diacylglycerol lipase); DAG (diacylglycerol); PLC (phospholipase C).

**Figure 3:**
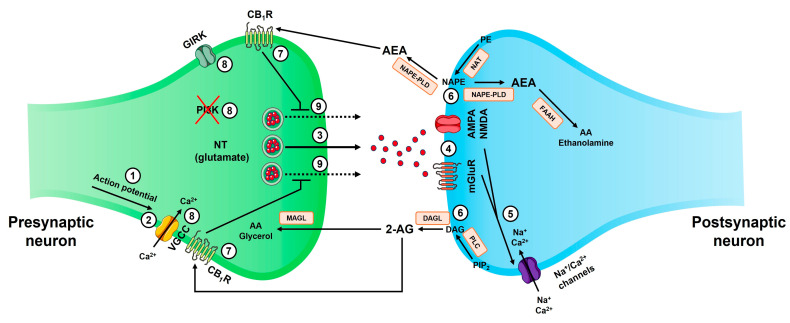
Diagram of endocannabinoid-mediated synaptic signaling. The steps involved in endocannabinoid action are as follows: **1** (the arrival of an action potential at the presynaptic terminal); **2** (activation of VGCCs, leading to Ca^2+^ influx); **3** (exocytosis of glutamate); **4** (interaction of glutamate with its corresponding receptors: AMPAR, NMDAR, and mGluR); **5** (entry of Na^+^ and Ca^2+^ ions into the postsynaptic terminal, resulting in its activation); **6** (synthesis and release of AEA and 2-AG); **7** (binding of AEA and 2-AG to CB_1_R); **8** (inhibition of GIRKs, VGCCs, and PI_3_K); **9** (inhibition of glutamate exocytosis). Abbreviations: CB_1_R (cannabinoid receptor 1); GIRK (G protein-coupled inwardly rectifying potassium channel); PI_3_K (phosphatidylinositide-3-kinase); NT (neurotransmitter); VGCC (voltage-gated calcium channel); AA (arachidonic acid); MAGL (monoacylglycerol lipase); 2-AG (2-arachidonoylglycerol); AEA (anandamide); PE (phosphatidylethanolamine); NAT (N-acetyltransferase); NAPE (N-acylphosphatidylethanolamine); NAPE-PLD (N-acyl phosphatidylethanolamine-specific phospholipase D); FAAH (fatty-acid amide hydrolase); AMPAR (α-amino-3-hydroxy-5-methyl-4-isoxazolepropionic acid receptor); NMDAR (N-methyl-D-aspartate receptor); mGluR (metabotropic glutamate receptor); PIP_2_ (phosphatidylinositol 4,5-bisphosphate); PLC (phospholipase C); DAG (diacylglycerol); DAGL (diacylglycerol lipase).

Endocannabinoids (Figure 1) are lipophilic compounds synthesized on demand from membrane lipids in response to receptor activation and increased intracellular Ca^2+^ levels [22]. Unlike classical neurotransmitters, which are pre-synthesized and stored in synaptic vesicles, endocannabinoids do not follow this process of vesicular storage and release [23]. Instead, endocannabinoids are synthesized in response to specific stimuli, such as the activation of postsynaptic receptors, and they exert their effects through retrograde signaling, traveling from the postsynaptic neuron to the presynaptic neuron [24] (Figure 2 and Figure 3).

Numerous synthetic pathways are involved in the production of endocannabinoids, with their importance varying across different tissues, developmental stages, and potentially under specific pathological conditions [22]. The synthesis of AEA (Figure 2 and Figure 3) begins with the conversion of phosphatidylethanolamine (PE) into N-arachidonoyl phosphatidylethanolamine (NAPE) by the Ca^2+^-dependent enzyme N-acyltransferase (NAT). AEA is then produced through the hydrolysis of NAPE by NAPE-PLD [25,26]. The primary pathway for 2-AG production (Figure 2 and Figure 3) relies on the Ca^2+^-dependent enzyme phospholipase C (PLC) and the activity of DAGL. PLC catalyzes the hydrolysis of phosphatidylinositol 4,5-bisphosphate (PIP_2_), resulting in the biosynthesis of diacylglycerol (DAG), which is then transformed into 2-AG by DAGL [25,27]. AEA and 2-AG are synthesized in response to many stimuli, including certain neurotransmitters [25,26,27] (Figure 2 and Figure 3).

However, the relative significance of these pathways can vary substantially depending on the cellular context, including alternative pathways, such as the α/β-hydrolase domain 4 (ABHD4) pathway for AEA synthesis or the phospholipase A_1_ (PLA_1_)-mediated route in 2-AG biosynthesis, becoming significant under certain circumstances [28,29]. This adaptability is governed by some factors, including Ca^2+^ signaling, enzyme phosphorylation, and transcriptional regulation, allowing for precise modulation in response to physiological demands [22]. On the other hand, the interrelationship of endocannabinoid synthesis with other lipid-signaling systems, including eicosanoid metabolism and phospholipid turnover, adds another layer of complexity to this dynamic system [30].

### 2.2. Endocannabinoid as a Retrograde Synaptic Messenger

Endocannabinoids are essential in retrograde signaling within the CNS, serving as essential modulators of synaptic transmission. Upon release, AEA and 2-AG move retrogradely across the synaptic cleft and bind to cannabinoid receptors, principally CB_1_R, located on the presynaptic terminal [31,32] (Figure 3). Activation of cannabinoid receptors leads to the suppression of neurotransmitter release, especially glutamate, thus impacting many neural processes, including pain perception [33,34]. This mechanism creates a feedback mechanism that allows postsynaptic neurons to regulate presynaptic neuron activity, thereby modulating the strength of synaptic connections (a process known as synaptic plasticity) [35,36]. Synaptic plasticity plays a key role in long-term potentiation (LTP) and long-term depression (LTD), both of which are essential for learning and memory [37,38] (Figure 3).

Research has indicated that disruptions in endocannabinoid signaling may result in aberrant synaptic plasticity, which might play a role in the onset and development of numerous pathologies, such as depression, epilepsy, anxiety, and fibromyalgia [39,40,41].

### 2.3. Cannabinoid Receptors

The CB_1_R gene (*CNR1*), first cloned by Matsuda et al. in 1990, encodes the CB_1_R [42]. In humans, *CNR1* is located on chromosome 6 (6q14-15) and includes four exons, with the fourth exon being the main coding sequence. In mice and rats, *CNR1* is situated on chromosomes 4 and 5, respectively [43] (Table 1). The human CB_1_R, a 53 kDa protein with 473 amino acids, is post-translationally modified into a 64 kDa glycosylated variant, which is more abundant than the non-glycosylated form (54 kDa) [44]. The CB_2_R gene (*CNR2*), initially cloned by Munro et al. in 1993, encodes the CB_2_R [18]. In humans, *CNR2* is located on chromosome 1 (1p36.11) [44], whereas in mice and rats, it is found on chromosomes 4 and 5, respectively [44] (Table 1). The human CB_2_R, composed of 360 amino acids (40 kDa), shares only 44% sequence homology with CB_1_R [45].

CB_1_R and CB_2_R are GPCRs that are activated by endocannabinoids, which are lipophilic ligands produced endogenously (Figure 1) [10,43,44]. CB_1_R is extensively expressed throughout the CNS [47,48,49,50]. Areas of high CB_1_R expression comprise the cerebral cortex (cingulate gyrus, prefrontal cortex, and hippocampus), periaqueductal gray, hypothalamus, cerebellum, amygdala, as well as the brainstem nuclei [47,48,49,50]. Moderate expression of CB_1_R has been observed in the spinal dorsal horn [51,52,53,54], and most recently, CB_1_R-positive fibers have been identified in the spinal ventral horn [55]. CB_1_R expression has been observed in the autonomous nervous system (ANS) and the enteric nervous system (ENS) [56,57,58,59]. CB_1_R has also been found in other organs, such as muscle [60], liver [61], adipose tissue [62], heart [63], pancreas [64], and lungs [71]. CB_2_R is strongly expressed in immune and pancreatic acinar cells [72,73,74,75], adipocytes [76], cardiomyocytes [77], endothelial cells [78], as well as in fibroblasts and osteoclasts [65,79]. 2-AG acts as a full agonist of CB_1_R and CB_2_R, whereas AEA functions as a partial agonist at both receptors [66] (Table 1).

Activation of CB_1_R and CB_2_R results in coupling with the G protein Gα_i/o_ (interactions with Gα_s_ and Gα_q_ have also been reported) [66,67,68,69], as well as β-arrestin recruitment [59], which activates many downstream effectors. Coupling with Gα_i/o_ results in the inhibition of adenylyl cyclase (AC), which significantly reduces the adenosine 3′,5′-cyclic monophosphate (cAMP) levels and/or activates the mitogen-activated protein kinase (MAPK) pathway [67,68,69,70]. The dissociation of Gβγ subunits from Gα_i/o_ triggers the activation of G protein-coupled inwardly rectifying potassium channels (GIRKs) and phosphatidylinositide-3-kinase (PI_3_K), accompanied by the strong inhibition of voltage-gated calcium channels (VGCCs) [67,68,69,70]. Cannabinoid receptors can also activate signaling through β-arrestin-dependent pathways, which involves the activation of ERK1/2 (extracellular signal-regulated kinases 1 and 2) and other MAPKs, receptor internalization and desensitization, and transcriptional regulation [80].

Finally, endocannabinoids interact with several orphan receptors [81]. In this context, various studies have debated whether GPR18, GPR55, and GPR119 should be classified as novel cannabinoid receptors [82,83,84]. These receptors possess pharmacological properties indicative of their potential inclusion in the ECS; however, their classification remains a subject of debate [85]. Ongoing research continues to explore their ligand specificity, downstream signaling pathways, and physiological implications, further accentuating their potential importance in cannabinoid pharmacology and therapeutic development [86].

### 2.4. Endocannabinoid Degradation

The degradation of endocannabinoids is a crucial process for regulating endocannabinoid signaling and maintaining homeostasis within the ECS [87]. The two principal endocannabinoids, AEA and 2-AG, are susceptible to enzymatic breakdown once they have completed their signaling roles [88]. AEA is mainly metabolized by FAAH, whereas 2-AG is hydrolyzed by MAGL [25,26,27]. These enzymes are strategically positioned within cellular membranes to facilitate the inactivation of endocannabinoids [31,32]. FAAH-mediated degradation of AEA (presynaptic terminal) results in the production of AA (arachidonic acid) and ethanolamine, whereas MAGL-mediated hydrolysis of 2-AG (postsynaptic terminal) leads to the formation of AA and glycerol [32,33] (Figure 2 and Figure 3).

Precise control of endocannabinoid degradation is essential for maintaining the stability of the ECS and preventing excessive or prolonged signaling, which can affect many physiological and pathological functions [88].

### 2.5. Endocannabinoid-Mediated Biological Processes

Upon binding to their specific receptors, endocannabinoids initiate sophisticated intracellular signaling cascades that impact neurotransmitter release [89], synaptic plasticity [90], and cellular homeostasis [91]. In the CNS, endocannabinoids are necessary for controlling synaptic transmission and neural circuit dynamics, thereby participating in regulating some cognitive functions [92], learning [93], and emotional responses [94]. In addition to their neuromodulatory functions, endocannabinoids are known for their neuroprotective properties, which involve the reduction of excitotoxicity, oxidative stress, and neuroinflammation [95,96,97,98]. These biological functions of endocannabinoids have broad implications for neurodegenerative diseases like Alzheimer’s and Parkinson’s [99,100].

Beyond the CNS, endocannabinoids modulate a diverse array of peripheral physiological systems, such as the immune, cardiovascular, respiratory, and digestive systems. In the immune system, endocannabinoids exert anti-inflammatory and immunomodulatory effects by regulating cytokine secretion, immune cell proliferation, and migration, thereby modulating immune homeostasis and response to disease conditions [101,102]. Within the cardiovascular system, endocannabinoids are involved in vasodilation, cardioprotection, and blood pressure regulation, highlighting their significance in cardiovascular well-being and perhaps their role in diseases such as hypertension and ischemic heart disease [103]. Conversely, some evidence suggests that endocannabinoids may induce bronchodilation by promoting the relaxation of airway smooth muscle, potentially offering therapeutic benefits for conditions including asthma and chronic obstructive pulmonary disease (COPD) [104]. Finally, endocannabinoids significantly reduce symptoms of gastrointestinal disorders like irritable bowel syndrome (IBS), inflammatory bowel disease (IBD), and nausea by suppressing pain and inflammation [105].

## 3. Relationship Between the ECS and Fibromyalgia

The notion that fibromyalgia might be linked to a deficiency in the ECS has attracted significant interest in recent years, providing a potential explanation for the symptomatology associated with this chronic pain condition [106]. This theory, called Clinical Endocannabinoid Deficiency (CECD), suggests that an underlying deficiency in the ECS may contribute to the development and persistence of fibromyalgia symptoms [107]. In fibromyalgia, dysfunction in the ECS is thought to lead to increased pain sensitivity and sleep disturbances. This hypothesis is supported by evidence indicating that cannabinoid-based treatments alleviate fibromyalgia symptoms, potentially through the restoration of deficient endocannabinoid tone [108]. Strand et al. carried out a comprehensive review of the existing literature, which proposed that cannabis might improve pain management and quality of life in individuals with fibromyalgia [15]. Moreover, the detection of cannabinoid receptors in fascial tissue, as demonstrated by Fede et al., suggests a potential mechanism by which the ECS may contribute to myofascial pain and the development of fibromyalgia symptoms [109]. Although the exact mechanisms are not yet fully understood, the exploration of the ECS’s role in fibromyalgia has been crucial in uncovering new ways of understanding the pathophysiology of this disease. Research into the role of the ECS in fibromyalgia has created several opportunities for developing new and effective treatments.

The involvement of the ECS in the biological oscillator is well-established, as it influences the sleep cycle [110]. The pineal gland produces melatonin and 2-AG in a circadian rhythm that is partially regulated through CB_2_R activation in the suprachiasmatic nucleus [111]. Endocannabinoids might also play a role in sleep onset, a factor that is particularly significant for individuals with fibromyalgia [112]. Alternatively, in young women, during the menstrual cycle, AEA levels decrease during the luteal phase and increase during the follicular phase. This variation is due to the progesterone-induced upregulation of FAAH during the luteal phase [113]. In a study of healthy women with normal menstrual cycles, a reduction in AEA was associated with increased sensitivity to pressure pain induced by an algometer during the luteal phase. Additionally, some participants showed variations in fibromyalgia diagnosis across the menstrual cycle, meeting the tender point criterion during the AEA-deficient luteal phase but not during the AEA-rich follicular phase [113].

On the other hand, the inflammatory degradation of connective tissues (a phenomenon observed in fibromyalgia patients) is blocked by CB_1_R activation [114]. Synovial cells, exposed to the pro-inflammatory cytokine TNF-α, secrete metalloproteinases, which play a role in the destruction of articular cartilage [115]. Moreover, research has shown that AEA attenuates articular cartilage destruction and inhibits nitric oxide-induced proteoglycan and collagen degradation [115].

Although differential expression of 421 genes has been documented in the development of fibromyalgia (e.g., *HDC*, *GATA2*, and *APBB2*), genome-wide expression profiling in patients with fibromyalgia did not reveal any alterations within the ECS [116]. Smith et al. identified the CB_1_R SNP (rs6454674) as a potential candidate for investigation in fibromyalgia patients [117]. However, a recent genotyping study conducted by Gerra et al. did not find statistically significant associations in the expression of CB_1_R or related SNPs between fibromyalgia patients and their controls [118]. When the fibromyalgia group was divided into subgroups considering their clinical features, a strong correlation was found between the CB_1_R SNP rs6454674 and depressive symptoms in fibromyalgia patients, compared to those without depression [118]. Additionally, polymorphisms in the *FAAH* gene, particularly rs324420, have been implicated in obesity and potentially pain sensitivity in fibromyalgia. This polymorphism might transform the metabolism of AEA, potentially influencing pain modulation [119,120]. These investigations suggest that these genetic variations affect the complex interplay between metabolic disorders, such as obesity, and chronic pain conditions, thereby providing valuable insights for developing personalized treatment strategies for fibromyalgia [8,121].

**Figure 4:**
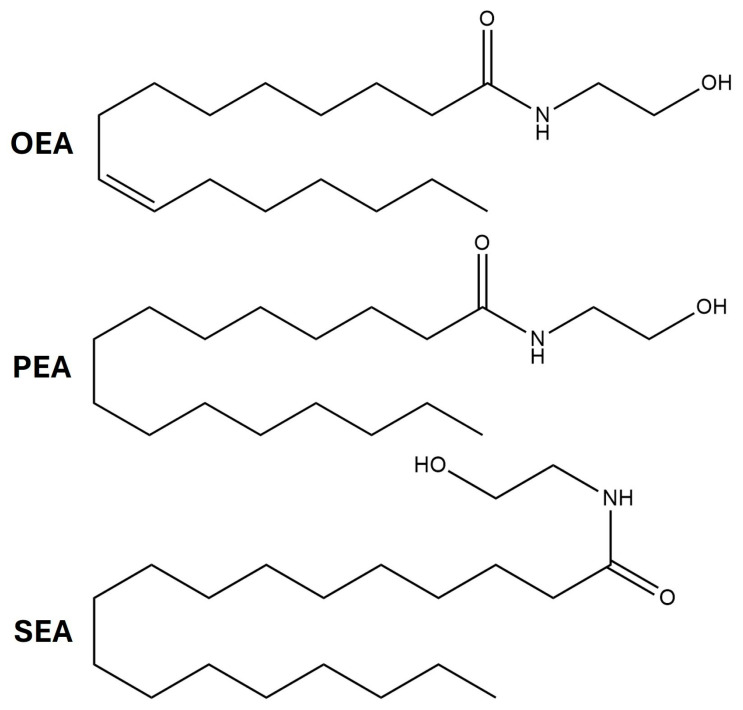
Chemical structures of OEA (oleoylethanolamine), PEA (palmitoylethanolamide), and SEA (N-stearoylethanolamine).

Alterations in circulating endocannabinoids (AEA and 2-AG) and related N-acylethanolamines (NAEs) in patients with fibromyalgia have been documented in some studies [122,123,124,125,126,127]. Researchers analyzed plasma levels of specific endocannabinoids and observed that plasma concentrations of AEA were significantly higher in patients with fibromyalgia compared to healthy controls [122,123]. Other studies [124,125] showed increased plasma contents of 2-AG, oleoylethanolamine (OEA), palmitoylethanolamine (PEA), and N-stearoylethanolamine (SEA) in fibromyalgia patients (Figure 4). The elevated levels of these lipids suggest a probable low-grade inflammatory component of fibromyalgia, considering that SEA is known for its anti-inflammatory effects [126]. OEA has shown antinociceptive effects in several animal models of inflammatory pain, independent of PPARα (peroxisome proliferator-activated receptor alpha) receptor activation [127]. In summary, the limited data available indicate elevated levels of 2-AG, AEA, and other related ethanolamines, suggesting a potential compensatory mechanism. However, further research is absolutely critical to validate this hypothesis and will require multiple longitudinal studies, large patient cohorts, and interventions such as FAAH inhibitors or CB_1_R agonists and/or antagonists to establish causality.

## 4. Cannabinoid-Based Therapies for Fibromyalgia

Cannabinoids interact with the ECS, which plays an essential role in regulating pain modulation in individuals with fibromyalgia. Emerging research shows that cannabinoid-based therapies might alleviate pain, improve sleep quality, and enhance overall well-being in individuals with fibromyalgia [128,129,130]. However, variability in dosing, lack of standardized formulations, and potential side effects constitute ongoing challenges. Several ethical considerations must be addressed when using cannabinoids to treat patients with fibromyalgia. Considering the limited long-term data available on the safety and efficacy of cannabinoids for fibromyalgia, it is essential to consider the potential for misuse, dependency, and adverse effects.

Further clinical studies are essential to establish optimal dosages, long-term safety, and efficacy, ensuring that cannabinoid therapies can be effectively integrated into treatment protocols for fibromyalgia.

### 4.1. Current Therapies

Clinical studies have shown that cannabinoid-based treatments may provide a viable therapeutic option for a broad spectrum of symptoms associated with fibromyalgia (such as pain, sleep issues, and mood disorders) [131,132]. Phytocannabinoids (extracted from *Cannabis sativa*) and synthetic cannabinoids have been rigorously examined as viable therapeutic avenues [133,134]. Cannabis has emerged as a promising treatment alternative for fibromyalgia, supported by many studies examining its effectiveness in managing various symptoms associated with the condition [128,129,130,135]. The anti-inflammatory and anxiolytic properties of CBD, in conjunction with the analgesic and muscle-relaxant properties of THC, might yield a synergistic effect in addressing the multifaceted symptoms inherent to this disorder [136,137].

Several studies published between 2011 and 2024 have investigated the potential benefits of cannabis for individuals with fibromyalgia. This timeframe was chosen to analyze the utilization of cannabinoids in fibromyalgia treatment, given significant scientific advancements, expanded clinical research, and regulatory developments. The increasing acceptance and legalization of cannabis in some countries have further facilitated research into its efficacy and safety. Moreover, ongoing shifts in healthcare practices during this period make it a critical timeframe for evaluating its impact on fibromyalgia management. In 2011, a study was conducted to evaluate the acute administration of cannabis in patients with fibromyalgia. The results demonstrated a significant reduction in pain and stiffness, improved relaxation, and an increase in drowsiness and overall well-being, observed two hours following cannabis use [138]. A survey conducted in Israel in 2018 revealed that 84% of patients with fibromyalgia reported cannabis use. Notably, 94% experienced pain relief, 93% observed improvements in sleep quality, 87% reported reductions in depressive symptoms, and 62% revealed improvement in anxiety [139]. Another research study conducted in 2019 showed that cannabis therapy led to a substantial reduction in pain intensity and an increase in quality of life for patients with fibromyalgia. This study displayed a 50% pain intensity in 81% of patients after six months of treatment [131]. In the same year, an experimental trial involving the inhalation of a preparation containing THC and CBD demonstrated low analgesic responses, with effects lasting a maximum of three hours [140]. This study confirms previous research indicating that the oral administration of THC significantly reduces pain levels associated with fibromyalgia [141]. Additionally, a 1:4 THC/CBD inhalation combination demonstrated substantial effects in a 2019 study involving fibromyalgia patients [142].

A systematic review published in 2021 analyzed 17 studies and concluded that cannabis-based medicines might be effective for pain relief and sleep improvement in fibromyalgia patients; however, the quality of evidence was considered moderate [143]. A 2024 study examined the combination of cannabis with oxycodone for pain management, finding that this combination reduced oxycodone consumption by 35% (without affecting the frequency of cannabis use) [144]. Another investigation from 2024 explored the efficacy of low-dose medical cannabis in treating fibromyalgia-related pain. The results revealed a substantial reduction in pain intensity and improvements in both physical and mental states for the majority of participants [145]. Additionally, a cohort study analyzing diverse cannabis-based medicinal formulations for fibromyalgia reported improvements across various outcome measures, including anxiety, sleep quality, and overall symptom severity [146].

Despite these promising findings, it is important to note that cannabis use for fibromyalgia is not without potential drawbacks. Several studies have reported side effects, such as dizziness, dry mouth, and gastrointestinal symptoms [131,140]. Moreover, the absence of randomized controlled trials has limited the capacity to comprehensively establish the efficacy and safety of cannabis-based medicinal products for the treatment of fibromyalgia. Moreover, patients should consult with healthcare professionals before considering cannabis as a treatment choice because their responses might vary significantly [15]. As research continues to progress, the potential of cannabis as a treatment choice for fibromyalgia remains an area of investigation, with ongoing efforts to determine optimal dosages, formulations, and administration techniques to maximize the benefits. Although promising results have emerged, it is essential to approach these findings with caution, recognizing that further study is necessary to establish definitive guidelines.

On the other hand, the use of cannabinoid derivatives (e.g., nabilone, dronabinol, and ajulemic acid) in the therapeutic management of fibromyalgia has produced positive results in recent years; this fact provides renewed optimism for individuals suffering from this condition. Nabilone, a synthetic cannabinoid, has evidenced considerable advantages in alleviating pain and enhancing functionality among patients afflicted with fibromyalgia [147,148]. In a randomized, double-blind, placebo-controlled trial, nabilone resulted in notable reductions in pain intensity, anxiety, and the overall consequences of fibromyalgia, as evaluated by the Fibromyalgia Impact Questionnaire (FIQ) [147,148]. Other observational trials showed that nabilone significantly reduced pain levels [149,150]. Ajulemic acid represents a novel approach in cannabinoid therapeutics; it possesses greater selectivity for CB_2_R over CB_1_R, in addition to activating PPARα, thereby reducing CNS side effects [151,152]. Ajulemic acid has demonstrated strong pain-relieving and anti-inflammatory effects in both animal studies and human trials, making it a promising candidate for the treatment of fibromyalgia [153]. Similar to ajulemic acid, dronabinol is a potential candidate for treating the painful symptoms of fibromyalgia due to its foundation in alleviating symptoms associated with neuropathic pain [154]. However, whereas these cannabinoid derivatives show potential, it is essential to recognize that the evidence supporting their use remains of low quality. Consequently, more comprehensive studies are necessary to definitively determine their efficacy and safety profiles.

Notwithstanding these encouraging findings, the utilization of cannabinoid therapies is not devoid of challenges. Numerous problems, such as variable patient responses, optimal dosing, potential side effects, and regulatory barriers, must be addressed through rigorous clinical trials and standardized guidelines [155,156]. Moreover, patient education and clinician training are crucial to ensure the safe and effective implementation of these therapies [157]. As the scientific understanding of fibromyalgia and the ECS continues to advance, cannabinoid-based treatments have the potential to become a key element in a personalized, multimodal approach to managing this complex condition [158].

### 4.2. Regulatory, Ethical, and Social Considerations in the Use of Cannabinoids in the Management of Fibromyalgia

The use of cannabinoids for the management of fibromyalgia is highly complex and influenced by regulatory, ethical, and social considerations [159,160,161]. A major regulatory challenge is the variation in the legal status of cannabis and its derivatives across different regions. In several countries, both medical and recreational cannabis have been legalized, establishing the necessary frameworks for controlled consumption. In contrast, in some countries, cannabis remains fully prohibited, with significant variation in the enforcement and penalties for possession or use [162]. This inconsistency creates a barrier to treatment for patients who could benefit from cannabinoid therapies but encounter substantial legal obstacles in accessing them [163]. For healthcare providers, the challenge lies in navigating these regulatory discrepancies while striving to deliver care that is legally compliant [164]. The lack of standardization complicates efforts to ensure that treatments for patients are reliable, effective, and safe [165,166]. Moreover, the uncertain regulatory landscape may obstruct the integration of cannabinoids into conventional medical practice despite the mounting evidence supporting their potential therapeutic benefits [165,166].

Ethical concerns arise, particularly due to the limited clinical evidence supporting the use of cannabinoids in the treatment of fibromyalgia [167]. While some investigations indicate that cannabinoids could significantly improve pain relief, sleep quality, and the quality of life for patients with fibromyalgia [131,132], this evidence is very far from being considered definitive. The limited nature of this research creates ethical dilemmas for clinicians who must make decisions about treatments that have not been adequately tested for long-term safety and efficacy [168]. This creates an intriguing dynamic where the imperative to alleviate patient suffering must be balanced with the principle of non-maleficence, compelling clinicians to avoid causing harm [168]. With the paucity of data on long-term risks of cannabinoids, especially for potential cognitive impairment, dependence, or other possible harm, clinicians might be very resistant to making these treatments part of standard practice. The ethical dilemma is further complicated by the fact that, in diseases like fibromyalgia, where conventional therapy often has little or no effect, there is a strong inclination toward innovative proposals. Finally, the decision to introduce cannabinoids hinges on balancing potential benefits, such as improvement in symptoms and quality of life, with long-term side effects that have yet to be fully recognized [169].

Finally, the increasing interest and advocacy for medical cannabis are leading to significant changes in societal attitudes [170,171]. With more healthcare consumers advocating for the use of cannabis in managing chronic conditions, there is increasing public support for expanding access to cannabinoid-based choices [172]. These shifts in public opinion have led to policy changes in various regions around the world, resulting in the legalization of medical cannabis in those countries where *Cannabis sativa* was previously prohibited [173]. The inclusion of cannabis into healthcare systems introduces numerous issues, heightening concerns about potential drug misuse and diversion while also presenting public health risks such as rising substance abuse, impaired driving, and other negative consequences [174]. The increased prevalence of cannabinoids has led to a higher demand for preventive measures to avoid exposures where the misuse of drugs may compromise individuals or those around them, particularly in the context of traffic accidents or colleagues in hazardous work environments [175]. The use of marijuana raises concerns over impaired driving, workplace safety, and addiction risk, highlighting the necessity for comprehensive public health initiatives and regulations to support responsible consumption [175].

## 5. Conclusions

The ECS is progressively recognized as a key regulator of various physiological processes, including pain perception, mood regulation, immune response, and inflammation, all of which are closely connected to the complex condition of fibromyalgia. Fibromyalgia is characterized by widespread chronic pain, fatigue, cognitive dysfunction, and other debilitating symptoms; however, its underlying mechanisms are not absolutely understood. Emerging research suggests that the ECS might play a significant role in the pathogenesis of fibromyalgia. Dysregulation of the ECS has been implicated as a contributing factor in this condition, with evidence pointing to altered levels of endocannabinoids or reduced function of CB_1_R and CB_2_R. Such disruptions could destabilize the natural ability to modulate pain and preserve homeostasis; as a result, this phenomenon could amplify pain sensitivity and intensify other symptoms typical of fibromyalgia.

Several studies have explored the potential of targeting the ECS for therapeutic purposes. Various studies suggest that cannabinoids or ECS modulators may alleviate pain, reduce inflammation, improve sleep quality, and enhance overall well-being in individuals with fibromyalgia. For example, treatments designed to amplify endocannabinoid signaling or directly activate cannabinoid receptors have exhibited promise in both preclinical and clinical contexts. However, the aforementioned studies examining the relationship between endocannabinoids and fibromyalgia suffer methodological limitations, like small sample sizes, observational study designs, or the absence of control groups. These factors significantly impact the reliability and generalizability of the findings, potentially leading to biased conclusions or an overestimation of the effects. Therefore, it is essential to explicitly acknowledge and critically evaluate these limitations to provide a more accurate interpretation of the evidence.

Despite these promising advancements, the exact correlation between ECS dysfunction and fibromyalgia remains not yet fully elucidated. The heterogeneity of fibromyalgia symptoms, coupled with the complexity of the ECS, necessitates further rigorous research to clarify these interactions. The integration of genetic profiling with clinical phenotyping offers a precision medicine approach that moves beyond the traditional “one-size-fits-all” treatment paradigm. By stratifying patients based on their symptoms, clinicians can refine diagnostic criteria and customize treatment plans to maximize therapeutic efficacy while reducing adverse effects. This approach might involve personalized dosing strategies, the selection of specific drugs (including those that enhance ECS activity) to target numerous molecular pathways, or the integration of non-pharmacological interventions such as lifestyle modifications, dietary adjustments, and cognitive–behavioral therapies, all customized to the patient’s individual genetic and metabolic profile. Future research should prioritize large-scale clinical trials to establish more definitive conclusions regarding the role of endocannabinoids in fibromyalgia.

## Figures and Tables

**Table 1:** Main characteristics of cannabinoid receptors. Abbreviations: CB_1_R (cannabinoid receptor 1) and CB_2_R (cannabinoid receptor 2).

Cannabinoid Receptor	Gene Localization	Tissue Localization	References
CB_1_R	Human: chromosome 6, Mouse: chromosome 4, Rat: chromosome 5	Cerebral cortex, basal ganglia, periaqueductal gray, hypothalamus, cerebellum, amygdala, brainstem medullary nuclei, spinal/ventral dorsal horn, autonomic nervous system, enteric nervous system, muscle, liver, adipose tissue, heart, pancreas, and lungs.	[42,43,44,46,47,48,49,50,51,52,53,54,55,56,57,58,59,60,61,62,63,64,65,66,67,68,69,70]
CB_2_R	Human: chromosome 6, Mouse: chromosome 4, Rat: chromosome 5	Immune cells, lymphoid tissues, pancreatic acinar cells, adipocytes, cardiomyocytes, endothelial cells, fibroblasts, and osteoclasts.	[18,43,45,65,66,67,68,69,70,71,72,73,74,75,76,77,78,79]

## Data Availability

Not applicable. No new data were generated.

## Abbreviations

The following abbreviations are used in this manuscript:

2-AG	2-arachidonoylglycerol
AA	Arachidonic acid
ABHD4	Abhydrolase domain containing 4, N-acyl phospholipase B
AC	Adenylate cyclase
ACR	American College of Rheumatology
AEA	Anandamide
AMPAR	α-amino-3-hydroxy-5-methyl-4-isoxazolepropionic acid receptor
ANS	Autonomic nervous system
APBB2	Amyloid beta precursor protein binding family B member 2
cAMP	Cyclic adenosine monophosphate
CB_1_R	Cannabinoid receptor 1 (protein)
CB_2_R	Cannabinoid receptor 2 (protein)
CECD	Clinical endocannabinoid deficiency
CNR1	Cannabinoid receptor 1 (gene)
CNR2	Cannabinoid receptor 2 (gene)
CNS	Central nervous system
COPD	Chronic obstructive pulmonary disease
DAG	Diacylglycerol
DAGL	Diacylglycerol lipase
ECS	Endocannabinoid system
EMBASE	Excerpta medica dataBASE
ENS	Enteric nervous system
ERK1/2	Extracellular signal-regulated kinases 1 and 2
FAAH	Fatty acid amide hydrolase
FIQ	Fibromyalgia Impact Questionnaire
GATA2	GATA-binding factor 2
GIRK	G protein-coupled inwardly rectifying potassium channel
GPCR	G protein-coupled receptor
GPR18	G protein-coupled receptor 18
GPR55	G protein-coupled receptor 55
GPR119	G protein-coupled receptor 119
HDC	Histidine decarboxylase
IBD	Inflammatory bowel disease
IBS	Irritable bowel syndrome
LTD	Long-term depression
LTP	Long-term potentiation
MAGL	Monoacylglycerol lipase
MAPK	Mitogen-activated protein kinase
MEDLINE	Medical literature analysis and retrieval system online
mGluR	Metabotropic glutamate receptor
NAE	N-acylethanolamine
NAPE	N-arachidonoyl phosphatidylethanolamine
NAPE-PLD	N-acyl phosphatidylethanolamine phospholipase D
NAT	N-acyltransferase
NMDAR	N-methyl-D-aspartate receptor
NT	Neurotransmitter
OEA	Oleoylethanolamine
PE	Phosphatidyl-ethanolamine
PEA	Palmitoylethanolamine
PI_3_K	Phosphatidylinositide-3-kinase
PIP_2_	Phosphatidylinositol 4,5-bisphosphate
PLA_1_	Phospholipase A_1_
PLC	Phospholipase C
PPARα	Peroxisome proliferator-activated receptor alpha
SEA	N-stearoylethanolamine
SNP	Single nucleotide polymorphism
THC	Tetrahydrocannabinol
TNF-α	Tumor necrosis factor alpha
VGCC	Voltage-gated calcium channel
